# A 10-Year Review of the Efficacy of Cranial Remolding Orthosis Treatment and Factors That Influence Outcomes for Infants with Isolated Deformational Plagiocephaly

**DOI:** 10.3390/children12081099

**Published:** 2025-08-21

**Authors:** Anna L. Trebilcock, Jill L. Findley, J. Suzanne Cherry, Jeffrey A. Kasparek, Melody M. Gordon, Stephen P. Beals, Timothy R. Littlefield

**Affiliations:** 1 Cranial Technologies, Inc., Tempe, AZ 85284, USA; jfindley@cranialtech.com (J.L.F.); tlittlefield@cranialtech.com (T.R.L.); 2 Phoenix Children’s Hospital Center for Cleft and Craniofacial Care, Phoenix, AZ 85013, USA

**Keywords:** deformational plagiocephaly, cranial remolding orthosis, helmet therapy, cranial deformity, CVAI, treatment outcomes

## Abstract

**Highlights:**

**What are the main findings?**

- Cranial remolding orthoses (CROs) are an effective, evidence-backed treatment for isolated deformational plagiocephaly in infants.
- Treatment efficacy has a negative relationship with infant age and severity rating, highlighting the need for earlier referrals to optimize treatment outcomes.

**What is the implication of the main finding?**

- This study provides useful information to pediatric health care providers regarding the significant clinical efficacy of CROs in treating plagiocephaly.
- Study findings help guide pediatric health care providers in the referral process by highlighting the main factors that impact CRO treatment outcomes in infants.

**Abstract:**

Background/objectives: The purpose of this study was to examine the overall efficacy and treatment outcomes of CROs in the treatment of isolated deformational plagiocephaly and investigate the variables that influence treatment efficacy. Methods: This was a 10-year retrospective review of N = 27, 990 infants with Isolated Deformational Plagiocephaly (IDP) who completed Cranial Remolding Orthosis (CRO) treatment between 3 and 18 months of age. Results: There was a significant overall mean change in CVAI(S) of −3.42 ± 0.011 (*p* < 0.001), and a significant improvement in CVAI(S) in all age groups, even in older babies (i.e., >11 months). Up to 96% of infants aged 4–6 months at initiation of treatment achieved a “good” or “great” outcome rating, and up to 77.6% of infants over 11 months exited with a similar outcome. The following were identified as significant predictors of greater change in CVAI(S): (1) younger entry age (*p* < 0.001, β = 0.01), (2) larger initial CVAI(S) scores (*p* <0.001, β = −0.43), (3) left plagiocephaly (*p* < 0.001, β = −0.36), and (4) and the absence of torticollis (*p* < 0.001, β = −0.17). Conclusions: CROs are an effective, research-supported treatment for IDP. Pediatric health care providers and parents should be aware of the efficacy of CRO therapy across age groups and severity ratings, the risk factors that may influence CRO outcomes, and the benefits of an early referral at a young age.

## 1. Introduction

Isolated deformational plagiocephaly (IDP) is a nonsynostotic skull deformity that is either congenital or acquired in nature, and characterized by unilateral parietooccipital flattening, ipsilateral anterior ear shift, ipsilateral forehead bossing, contralateral forehead flattening, and contralateral posterior parietooccipital bossing [1]. Facial asymmetry is also a common feature of IDP which may result in eye, cheek, nose, jaw, or chin misalignment [2]. Deformational forces conducive to the development of plagiocephaly may result from in utero positioning, extended periods of supine infant placement (e.g., supine sleeping, prolonged time in car seats or other infant positioning devices), and neck muscle tightness with a cervical rotation preference (i.e., torticollis) [3,4].

CROs are a custom-made helmet designed to gently correct skull-shape deformities. Multiple studies have demonstrated their efficacy and reliability for treating deformational plagiocephaly [5,6,7,8,9,10,11,12,13,14,15,16,17,18,19,20,21]. Previous research has revealed that initial severity and younger age at onset of treatment are associated with better treatment outcomes [12,14,15,16,21,22,23,24,25,26]. A systematic review and evidence-based guideline paper by the Congress of Neurological Surgeons (CNS) concluded that infants who present with a more severe cranial deformity and begin treatment earlier in infancy will typically have a greater correction to their head shape [27]. Even with professional consensus in this area, additional research with a larger sample size is needed to examine all the different variables that may influence treatment outcomes and investigate the efficacy of treatment among different age groups and severity ratings.

A recent systematic review considered the impact of nine different clinical factors impacting CRO efficacy and treatment outcomes [28]. The authors found that treatment success correlated with the patient’s age at the onset of treatment, adherence to CRO usage, and the initial severity of the deformity. Further, they found no significant relationship between the presence of torticollis or gestational age on final outcomes [28]. Another systematic review found the effectiveness of helmet therapy in correcting cranial deformities surpasses alternative methods currently available (e.g., repositioning, physical therapy) [29]. They stipulated that CRO treatment outcomes are influenced by factors such as the age of patients at initiation of treatment, the duration of treatment, and the severity of the presenting deformity.

Kelly et al. examined the factors that impact treatment outcomes with isolated deformational brachycephaly (IDB) and found that entry age impacts treatment outcomes and total treatment time [11]. However, IDP has different clinical presentations than brachycephaly, and plagiocephaly often involves other concomitant factors (e.g., torticollis/ neck muscle involvement). Several studies have discussed the impact of individual factors on the treatment of plagiocephaly such as entry age [12,14,15,16,21,22,23,24,25,26], type of insurance coverage [13,30], multiple births [31], presence of torticollis [32,33], and deformational severity [14,24,34].

However, previous research has been limited by small sample sizes and does not explore the full range of potential treatment factors. This is the first study to examine the overall efficacy and treatment outcomes of CROs in the treatment of isolated deformational plagiocephaly and investigate the variables that influence efficacy of treatment in the context of a very large sample size.

## 2. Materials and Methods

### 2.1. Definitions

The need for standardized language to document infant skull dysmorphology has been recognized [35,36]. The anthropometric measurements commonly used to quantify infant head shape include the Cephalic Index (CI), Cranial Vault Asymmetry (CVA), and Cranial Vault Asymmetry Index (CVAI). CI is defined as cranial width/cranial length ×100, where cranial width represents the maximum distance of the head.

CVA is defined as the difference between the longer (Diagonal B) and shorter diagonal (Diagonal A). Diagonal B represents the distance between the most prominent frontozygomatic point and the most prominent occipital point. Diagonal A represents the distance from the most depressed frontozygomatic point and the most depressed occipital point. The CVAI was originally described by Loveday and de Chalain using the shorter diagonal as the denominator and was later denoted as CVAI(S) by Miyabayashi [36], to specify that the denominator of the formula represents the shorter of the two oblique diagonals. For the purposes of this paper, we used CVAI(S) as reported by Loveday and de Chalain which is defined as [37]:

(Diagonal_Longer_ − Diagonal_Shorter_)/Diagonal_Shorter_ × 100

Others have modified this formula to divide CVA by the longer of the two diagonals, where this formula is denoted as CVAI(L) [36]. Miyabayashi found in a systematic review of CVAI usage among research papers, that 20/30 qualifying articles used the CVAI(S) and 10/30 used the CVAI(L); however, industry efforts are now being made to standardize this formula across all providers/researchers. For the purposes of this study, IDP was defined by the following parameters: (a) a deformational head shape of non-synostotic and non-syndromic origin, (b) CI < 90 and >75, and (c) CVAI(S) greater than 3.5.

### 2.2. Study Design

This study was a retrospective review of infants with isolated deformational plagiocephaly who completed and complied with treatment with a CRO (DOC Band^®^, Cranial Technologies, Tempe, AZ, USA) throughout the United States from 1 July 2014–25 March 2024. The recommendations for treatment were based upon the initial physician referral due to concerns about the infant’s head shape, a comprehensive medical history and clinical exam, a 3-dimensional digital image review of skull morphology, and consideration of the physician and parent treatment objectives and expectations. The CRO is a prescription only, custom-made medical device comprising an interior layer of closed-cell polyethylene foam and an exterior thermoplastic copolymer shell (polypropylene-polyethylene blend). The device is designed for each infant’s unique deformity, applying a gentle holding pressure on the prominent aspects of skull, while providing room for growth in the adjacent flattened areas. Trained clinicians specializing in the care of infants with plagiocephaly adjust the orthosis over time to monitor growth, correction and ensure optimal outcomes. At the patient’s exit appointment, clinicians determined compliance, defined as 23 h/day wear, on a binary yes/no parent-reported variable. “Yes” was defined as compliant with the 23 h/day wear time protocol throughout treatment.

### 2.3. Data Source

**Figure 1:**
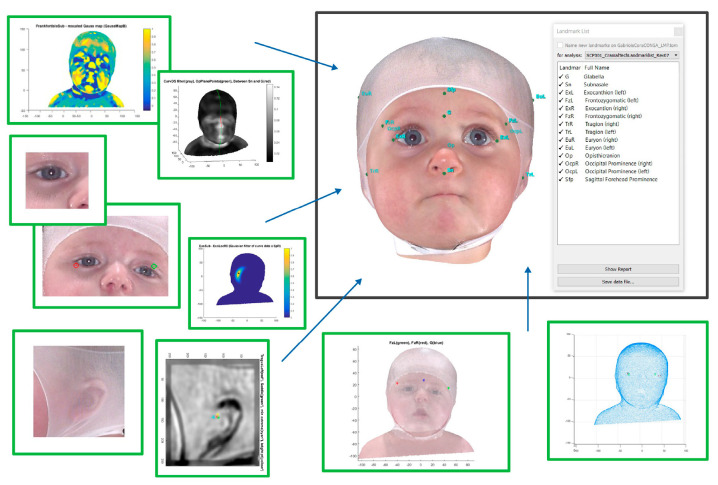
Multiple algorithms utilizing various texture maps, curvature, and curvedness are used to precisely identify all key landmarks used for measurement from the original 3D DSi^®^ image.

Patient data was queried from Cranial Technologies’ electronic health record (EHR) system (Intergy-Greenway Health, Tampa, FL, USA) and its 3-dimensional Digital Surface Imaging^®^ (DSi^®^) system using Microsoft SQL Server Management Studio (version 15.0.18424.0, SSMS, Redmond, WA, USA). The DSi is a stereophotogrammetry-based 3D imaging system designed specifically for infants with cranial deformation. The system employs triangulated digital cameras to capture a complete 3D image in less than a second. It is a photographic-based system that does not expose the infant to any coherent (i.e., laser) light, or radiation that is found in X-rays or CT scans [38,39]. These 3D head shapes are initially processed through a proprietary program (Landmarks 3D^®^, Cranial Technologies, Inc., Tempe, AZ, USA) which analytically identifies all key cranial landmarks which are then used to calculate anthropometric measures (Figure 1). In order to design and manufacture the cranial orthosis, the 3D image is imported into a proprietary Machine Learning (ML) based software application (Sentient3D^®^, Cranial Technologies, Inc., Tempe, AZ, USA) first introduced in 2005. Using the large datasets captured by the DSi system, a support vector machine (SVM) based ML application was trained to replicate the hand rectification and design process of the creator of the DOC Band and her decades of clinical experience. This program has learned how to examine the infant’s original deformity, determines where to place the mild holds, and how much growth room should be provided for the patient. The software is validated, under strict version and change control procedures, and each output is reviewed and examined by experts in our digital technologies department. The software is not in a state of continuous training as many individuals may incorrectly assume because, as frequently reported on social media, this can result in AI hallucination, loss of control, and inaccuracy of outputs.

Collection and analysis of the retrospective data did not involve direct contact with patients, their families, or their physicians. Institutional Review Board (IRB) approval was obtained in November 2023 from Argus IRB and a waiver of informed consent was granted.

The demographic variables collected in this study included consult age, CRO entry age, lapse in days from consult to CRO entry, treatment duration, need for a helmet remake, sex, ear shift, eye orbit shift, head height discrepancy, eye fissure narrowing, presence of torticollis, plurality (i.e., multiple birth infants), laterality of plagiocephaly, need for multiple bands, instruction to parents/guardians at consultation in head/body repositioning techniques, presence of a cranial hematoma, treatment with more than one CRO, initial and final CVAI(S), change in CVAI(S), initial and final CVA, change in CVA, initial and final CI, change in CI, initial and final head circumference, and change in head circumference.

### 2.4. Subject Identification

Patients were included in this study if they met the IDP anthropometric parameters (initial CVAI(S) greater than 3.5 and initial CI greater than 75% but less than 90%) and also had: an entry age between 3 and 18 months, a gestational age >37 weeks, complete treatment records, and overall compliance with the treatment protocol (23 h/day use). Patients were excluded from the study if they had: (a) previous treatment with a different CRO, (b) craniosynostosis/syndromic head shape, (c) a gestational age of <37 weeks, (d) non-compliance with the treatment protocol, (e) record duplications, (f) treatment lapse >30 days, (g) incomplete data records, and (h) data entry/validation errors (Figure 1). Patients were subsequently sub grouped by entry age (≤4 months, 4 to ≤6 months, 6 to ≤8 months, 8 to ≤11 months, and >11 months) and initial severity rating: mild (3.5 < CVAI(S) ≤ 6.25), moderate (6.25 < CVAI(S) ≤ 8.75), severe (8.75 < CVAI(S) ≤ 11), and very severe (CVAI(S) > 11).

### 2.5. Statistical Analysis

The mean change in CVAI(S) was the primary outcome measure in this study. Paired *t*-tests and ANOVA were used to evaluate whether there was significant change in infant’s CVAI(S) entry and exit measurements for the overall sample size and for each of the five age groups (<4 months, ≥4 to <6, ≥6 to <8, ≥8 to <11, and ≥11). Multiple regression was used to examine change in CVAI(S) with the following predictor variables: (1) initial CVAI(S); (2) entry age; (3) sex; (4) torticollis; (5) band remake; (6) laterality of plagiocephaly, and (7) child of a multiple birth. All tests were two-tailed and a *p*-value of <0.05 was set to indicate statistical significance. The data was analyzed using the statistical program R 4.3.1 (The R Foundation, Vienna, AT, USA) and Excel (version 2507, Microsoft, Redmond WA, USA). Outlying data points were excluded from the dataset if there were more than three standard deviations above or below the mean.

## 3. Results

### 3.1. Sample Demographics

**Figure 2:**
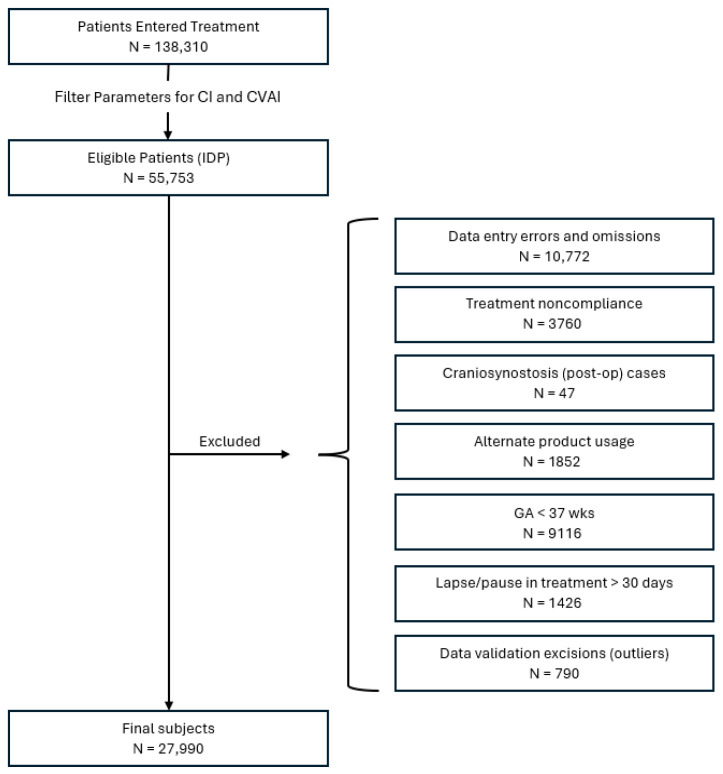
Flow Chart Depicting Patients Included in the Sample.

N = 138,310 infants entered treatment between July 2014 and March 2024, and of those cases, a final sample of N = 27,990 infants met both the inclusion criteria for IDP and completed CRO treatment (Figure 2). There were 18,412 males and 9578 females represented in the sample. The sample was comprising N = 1358 patients in the <4 month category, N = 13,249 patients in the ≥4 to <6 month category, N = 8617 patients in the ≥6 to <8 month category, N = 3866 patients in the ≥8 to <10 category, and N = 900 patients in the ≥11 month category. The age at entry into treatment ranged from 3 to 18 months, with a mean of 6.5 months. Treatment duration ranged from 25 days to 7.7 months, with a mean of 3 months. Younger babies had significantly shorter treatment durations (Table 1). The mean change in CVAI ranged from −16.02 to 1.18 with a mean change across all age groups of −3.42 ± 1.87 (S.D) (Table 1). Study subjects were further categorized by age at initiation of treatment and initial severity (Table 2).

### 3.2. Change in CVAI(S) and CI

Paired *t*-tests revealed significant improvement in infants’ pre-treatment versus post-treatment CVAI(S) scores across all age groups (*p* < 0.001). The pretreatment CVAI(S) ranged from 3.52 to 22.66 with an overall mean of 8.33 +/− 2.81 (S.D) and the posttreatment CVAI(S) ranged from 0.0 to 11.52 with an overall mean of 4.91 +/− 2.07 (S.D). The overall mean change in CVAI(S) was −3.420 ± 1.87 (S.D). An ANOVA test for differences between groups showed that there were significant differences among groups (F = 841.73, *p* < 0.001). Single Factor ANOVA with Bonferroni corrections to *p*-values confirmed statistical significance and independence within and between groups for mean CVAI(S). Paired t-tests also revealed that there was a statistically significant mean reduction in CI across all age groups (mean initial CI = 85.54 ± 0.02 (S.E.) and final CI = 83.69 ± 0.019 (S.E.)) and also within each of the five age groups (*p* < 0.001).

### 3.3. Treatment Duration

ANOVA and post hoc *t*-tests with Bonferroni corrections demonstrated that treatment durations were statistically different between each age group (*p* < 0.001) indicating that younger babies had shorter treatment durations. For example, babies who started treatment between 3 and 4 months of age had an average treatment duration of 10 weeks, while babies who started treatment at 11–18 months of age had an average treatment duration of 16 weeks (Table 1). The overall average treatment duration was approximately 3 months.

**Figure 3:**
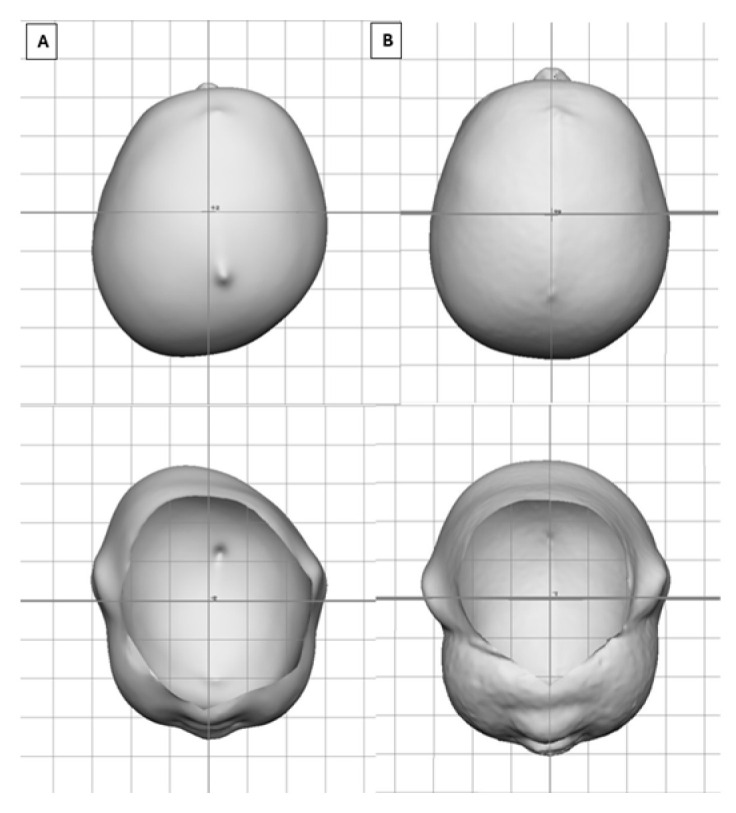
Vertex view of a 3.5-month-old after 1.7 months of treatment. (**A**) Entry CVAI(S) of 12.7. (**B**) Exit CVAI(S) of 1.5.

**Figure 4:**
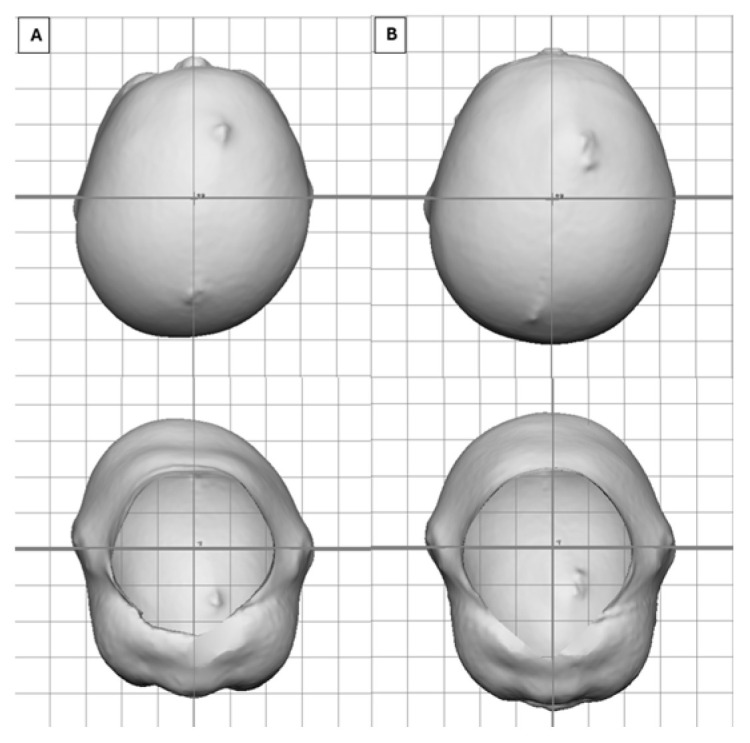
Vertex view of a 7.6-month-old after 4.5 months of treatment. (**A**) Entry CVAI(S) of 9.8. (**B**) Exit CVAI(S) of 3.4.

Figure 3 depicts a very severe infant (CVAI(s) = 12.7) who entered treatment at 3.5 months of age who saw an overall change in CVAI of −11.2 (88%) in 1.7 months of treatment, whereas Figure 4 depicts a severe infant (CVAI(s) = 9.8) who entered treatment at 7.5 months of age and saw an overall change in CVAI of −6.4 (65%) in 4.5 months of treatment.

### 3.4. Provider-Rated Outcome of Treatment

At exit from treatment, providers rated the outcome of treatment using a 4-point outcome rating scale comprising poor/fair/good/great outcome. For infants who started CRO treatment between three to six months of age, 93.7–96.6% of infants achieved a “good” or “great” outcome rating by their treating clinician at the time of exit from treatment, regardless of their initial severity rating. For babies who were older than six months of age, the percentage of babies with “good” or “great” outcomes decreased as severity and age increased. For example, 55.1% of babies with a severe head shape who were greater than 11 months of age at entry into treatment obtained a “good” or “great” outcome (Table 3).

### 3.5. Pretreatment Versus Posttreatment Classification of Head Shape

Of the 27,990 infants in this retrospective study, 4842 (17.3%) infants were anthropometrically classified as very severe plagiocephaly (CVAI(S) > 11) at the beginning of treatment. Of those, 2% (97) finished treatment in the normal category; 26.4% (1277/4842) finished as mild; 48.8% (2361/4842) were moderate; 21.4% (1037/4842) finished as severe; and only 1.5% (70/4842) of cases remained in the very severe category. Among these 4842 infants who were initially classified as having a very severe deformity at the initiation of treatment, 98.6% (4772/4842) improved by one or more categories of severity by the treatment’s conclusion, with 77.1% (3735/4842) having improved by two or more categories. Overall, 87.6% of the total infants in the sample (24,521/27,990) improved by one category of severity or more following treatment with a CRO, and 75.2% (21,045/27,990) exited treatment with a “normal-to-mild” classification (Table 4).

### 3.6. Clinical Predictive Factors Associated with Change in CVAI(S)

The initial standard multiple regression model included the following factors: (1) initial CVAI(S); (2) entry age; (3) sex; (4) torticollis; (5) band remake; (6) laterality of plagiocephaly; (7) infant of a multiple birth. The final model included only the statistically significant variables and revealed that the following factors were significant predictors of greater change in CVAI(S): (1) age (in days) at initiation of treatment (*p* < 0.001, β = 0.01), (2) initial CVAI(S) (*p* < 0.001, β = −0.43), (3) left plagiocephaly (*p* < 0.001, β = −0.36), and (4) and the absence of torticollis (*p* < 0.001, β = −0.17) (Table 5).

## 4. Discussion

This study of 27,990 patients is the largest retrospective examination in support of CRO treatment efficacy for patients between 3 and 18 months of age with IDP to date. The findings from this study are consistent with previous studies which indicated that CROs are effective in treating deformational plagiocephaly [5,6,7,8,9,10,11,12,13,14,15,16,17,18,19,20,21], and results demonstrate that an earlier entry age is associated with more favorable outcomes. Hauc et al. recognized the need for prompt referral, as treatment outcomes depend heavily on entry age [13]. Cevik et al. also recommended early onset of treatment prior to 6 months old to increase CRO effectiveness [25]. Similarly, Graham et al. found that treatment times increased with severity and age at start of treatment [14]. Current consensus standards developed by the Congress of Neurological Surgeons (CNS) with the American Academy of Neurological Surgeons (AANS) and endorsed by the American Academy of Pediatrics (AAP), affirm that infants who begin treatment earlier in infancy will typically have better outcomes and have a higher chance of achieving normalized head shape [40].

This study revealed that 69.7% of infants had right plagiocephaly (right parietal-occipital flattening) and 30.3% had left plagiocephaly (left parietal-occipital flattening). The prevalence of right versus left plagiocephaly from our results aligns with the findings from previous studies, which also report a higher prevalence of right-sided plagiocephaly [41,42,43,44,45,46,47,48].

Often, treatment with CROs is not recommended for babies with mild head shapes. However, a recommendation for CRO treatment is based upon a comprehensive clinical evaluation of a head shape and assessment of cranial anthropometric measurements. The babies with mild head shapes included in this study may have had notable 3-dimensional morphological skull deformities (e.g., ear shift, facial asymmetry) which may not have been adequately captured in the CVA, CVAI, or CI values. Rather, these cases require more specific anthropometric measurements such as the orbitaltragial (OBTA) and skull base asymmetries (SBA) as originally described by Ripley et al. [42,49]. Certain 3-dimensional skull deformities (e.g., head height asymmetry, posterior skull height, forehead sloping) cannot be captured with standard linear anthropometric measurements.

CRO treatment for isolated deformational plagiocephaly led to significant improvements in CVAI(S) across all age groups and severity categories. The multiple regression analysis revealed that a younger age at initiation of treatment, larger initial CVAI(S) scores, left plagiocephaly, and the absence of torticollis were found to be clinically significant predictors of greater change in CVAI(S). Graham et al. found that presenting age, presenting severity, and the presence of torticollis influenced treatment durations; however, their findings indicated that the presence of torticollis did not have a significant effect on final CVAI [14].

It is possible that the role of torticollis/neck muscle involvement in plagiocephaly may have been underestimated in this study, as the data capture method for recording the presence of torticollis occurs during a medical history review with the parent(s). Torticollis may be present at the consultation even if the physician has not yet diagnosed torticollis [50]. Previous research has shown that torticollis and plagiocephaly may coexist in 90% of infants with the deformity [51]. Best treatment outcomes likely occur when the neck muscle involvement is appropriately managed with concurrent therapeutic exercise, repositioning, and physical therapy intervention. The finding that babies with left plagiocephaly experienced greater change in CVAI(S) scores was unexpected and may be related to differences in neck muscle involvement in left versus right plagiocephaly [50]. This is an area that warrants further investigation.

The primary complications encountered in this cohort were red spots and heat rash. The exact incidence is difficult to quantify, as there are many variables and reporting mechanisms that influence this figure. However, the prevalence of red spots typically falls between 20 and 30%. No serious adverse events were reported.

Study findings support that early identification and management of deformational plagiocephaly leads to more optimal outcomes and reduced treatment time. Delays in treatment must be accounted for when making a referral for a CRO, as scheduling and insurance approval can occasionally take 4–6 weeks. Furthermore, prompt intervention is indicated particularly when certain factors are present such as torticollis, right-sided plagiocephaly, a CVAI(S) >3.5, and an entry age >6 months.

### Limitations

The findings of this study should be considered in the context of multiple limitations. This was not a randomized controlled trial (RCT), but rather a retrospective cohort review of patients who were full-term, compliant with treatment protocol, and completed a full course of treatment. The compliance measurement was based on subjective, parent-reported feedback which is often imprecise and biased [52,53,54]. The inclusion of a more objective means of determining compliance, such as the usage data from a compliance sensor, would be a better method for reporting wear times and identifying compliance issues. Several studies have reported on the use of compliance sensors to help accurately monitor patient wear times, which could be a helpful tool used to improve patient outcomes [52,53,54]. A further limitation is that this study used linear anthropometric measurements as its main outcome measure, and these measurements are unable to quantify the 3D nature of a skull shape [2,35]. Additionally, most infants have a clinical presentation of some degree of a combinational head shape (a mix of brachycephaly and plagiocephaly). In this study, N = 54,752 patients were excluded from the sample for having a combinational head shape. Therefore, the results from this study have limited generalizability to CRO use with other types of head shape deformities. Furthermore, the results from the study pertain to only one type of CRO, and the outcome data from other types of CROs were not included in this study.

Several areas of this research warrant further investigation. Future studies could be designed to examine the diagnosis and treatment of combinational head shapes (an area that has received little attention in the literature), assess patient compliance and the factors that influence wear time, investigate the concurrence of skull base/facial asymmetry particularly in the presence of torticollis/neck muscle involvement, examine the duration of treatment and outcome differences for infants with left versus right plagiocephaly, and develop a predictive model to model final CVAI(S) outcomes. The use of predictive analytics may contribute to establishing realistic CRO treatment outcome expectations. Infants with IDP across all age groups were also found to have significant reductions in their CIs with the use of a CRO.

## 5. Conclusions

This study provides useful information regarding the significant clinical efficacy of CROs in treating plagiocephaly across age groups and severity ratings and identification of clinical factors that impact CRO treatment outcomes for isolated deformational plagiocephaly. Consideration of these factors can be used to guide assessment, management, treatment planning, and goal setting for CRO treatment. Successful treatment outcomes can be achieved in older infants, and treatment expectations should be managed regarding expected treatment durations and final outcomes based on age, presenting initial CVAI(S), presence of torticollis, and laterality of plagiocephaly. The youngest babies (3–4-month age group) showed the most successful outcomes, largest effect size, and shortest treatment durations, whereas the older babies (11-month-old or greater) had less successful outcomes, the smallest effect size, and longer treatment durations. These results emphasize that early referral to treatment at 3 months of age is beneficial, as delays between evaluation and treatment initiation are common due to the time needed for scheduling and insurance review.

Pediatric health care providers and parents should be aware of the efficacy of CRO therapy across age groups and severity ratings, the risk factors that may influence CRO outcomes, the benefits of an early referral at a young age, and that CROs are an effective, research-supported treatment for IDP that has demonstrated favorable outcomes for infants.

## Figures and Tables

**Table 1:** IDP Patient Demographics Categorized Overall and by Entry Age.

Variable	All, N = 27,990	<4, N = 1358	≥4 to <6, N = 13,249	≥6 to <8, N = 8617	≥8 to <11, N = 2866	≥11, N = 900
Consult age (days)	162	n/a	n/a	n/a	n/a	n/a
Entry age (days)	192	n/a	n/a	n/a	n/a	n/a
Consult to Entry Lapse (days)	30	25	26	34	38	40
Duration (weeks) ***	12.9	10	11.4	14	15	15.9
Band Remake	976	84	547	222	105	18
Fit Modification	7154	411	3281	2258	969	235
Male	18,412	948	8774	5593	2489	608
Female	9578	410	4475	3024	1377	292
Frontal Flattening (Left)	18,939	954	9130	5793	2488	574
Frontal Flattening (Right)	8253	382	3821	2544	1224	282
Anterior Ear Shift (Left)	7982	380	3698	2456	1172	276
Anterior Ear Shift (Right)	17,735	914	8630	5382	2283	526
Anterior Orbit Shift (Left)	6311	315	2948	1931	908	209
Anterior Orbit Shift (Right)	13,815	743	6789	4137	1753	393
Head Height (Left)	6978	353	3283	2141	979	222
Head Height (Right)	15,355	836	7595	4607	1891	426
Eye Fissure Narrowed (Left)	6995	409	3435	2059	894	198
Eye Fissure Narrowed (Right)	3533	181	1690	1041	494	127
Limited Neck ROM (Includes Torticollis)	13,550	812	6989	3954	1498	297
Torticollis	7331	427	3790	2139	802	173
Multiple Birth	1186	53	579	369	156	29
Plagiocephaly (Left Sided)	8492	393	3896	2645	1267	291
Plagiocephaly (Right Sided)	19,498	965	9353	5972	2599	609
Repositioning Techniques	13,735	923	7362	3911	1336	203
Multiple Bands	3197	318	2040	662	161	16
Hematoma	248	14	117	71	38	8
Initial CVAI(S)	8.327	9.569	8.723	7.968	7.507	7.585
Final CVAI(S)	4.908	4.814	4.864	4.908	4.960	5.463
Mean Change in CVAI(S) ***	−3.420	−4.755	−3.859	−3.060	−2.547	−2.122
Initial CVA (mm)	11.143	12.104	11.457	10.820	10.495	10.943
Final CVA (mm)	7.089	6.728	6.939	7.150	7.335	8.211
Mean Change in CVA (mm)	−4.054	−5.376	−4.518	−3.670	−3.160	−2.732
Initial C.I.	85.543	85.280	85.648	85.575	85.359	84.878
Final C.I.	83.688	84.091	83.827	83.552	83.447	83.378
Change in C.I. ***	−1.855	−1.189	−1.821	−2.023	−1.912	−1.500
Consult Circumference (mm)	431.097	409.244	423.305	435.793	447.713	462.428
Exit Circumference (mm)	452.724	437.047	446.658	456.899	464.743	474.082
Change in Circumference (mm) ***	21.628	27.803	23.353	21.106	17.030	11.653

*** significant at *p* < 0.001; highlighted with grey background color.

**Table 2:** Classification of subjects by age at initiation of treatment and initial plagiocephaly severity (CVAI).

Entry Age	Mild		Moderate		Severe		Very Severe	
	≥3.5 to ≤6.25		>6.25 to ≤8.75		>8.75 to ≤11.0		>11.0	
<4	204	(2.8%)	369	(4.0%)	376	(5.8%)	409	(8.5%)
≥4 to <6	3017	(40.8%)	4049	(43.8%)	3377	(51.8%)	2806	(58.0%)
≥6 to <8	2509	(33.9%)	3045	(33.0%)	1897	(29.1%)	1166	(24.1%)
≥8 to <11	1359	(18.4%)	1444	(15.6%)	686	(10.5%)	377	(7.8%)
≥11	308	(4.2%)	330	(3.6%)	178	(2.7%)	84	(1.7%)
Total	7397		9237		6514		4842	

**Table 3:** Clinician-reported Rating of Good or Great Outcome based on Age and Initial Severity.

Entry Age	Mild		Moderate		Severe		Very Severe	
	(≥3.5 to ≤6.25)		>6.25 to ≤8.75		>8.75 to ≤11.0		>11.0	
<4	197	(96.6%)	358	(97.0%)	367	(97.6%)	392	(95.8%)
≥4 to <6	2881	(95.5%)	3871	(95.6%)	3221	(95.4%)	2628	(93.7%)
≥6 to <8	2351	(93.7%)	2784	(91.4%)	1672	(88.1%)	995	(85.3%)
≥8 to <11	1199	(88.2%)	1182	(81.9%)	535	(78.0%)	258	(68.4%)
≥11	239	(77.6%)	228	(69.1%)	98	(55.1%)	41	(48.8%)
Total	6867		8423		5893		4314	

**Table 4:** Pretreatment versus Posttreatment Classification of Severity.

Pretreatment Classification			Posttreatment Classification									
	Total		WNL		Mild		Moderate		Severe		Very Severe	
			(<3.25)		(≥3.5 to ≤6.25)		(>6.25 to ≤8.75)		(>8.75 to ≤11.0)		(>11.0)	
Mild (≥3.5 to ≤6.25)	7397	(26.4%)	5008	(67.7%)	2384	(32.2%)	5	(0.1%)	0	(0.0%)	0	(0.0%)
Moderate (>6.25 to ≤8.75)	9237	(33.0%)	2194	(23.8%)	6188	(67.0%)	853	(9.2%)	2	(0.0%)	0	(0.0%)
Severe (>8.75 to ≤11.0)	6514	(23.3%)	507	(7.8%)	3390	(52.0%)	2462	(37.8%)	153	(2.4%)	2	(0.0%)
Very Severe (>11.0)	4842	(17.3%)	97	(2.0%)	1277	(26.4%)	2361	(48.8%)	1037	(21.4%)	70	(1.5%)

**Table 5:** Multiple Linear Regression Analysis of Clinical Predictor Factors on Efficacy as Measured by Mean Change in CVAI(S)—Final Model.

Final Multiple Linear Regression Model						
Regression Statistics						
Multiple R					0.713	
R Square					0.508	
Adjusted R Square					0.508	
Standard Error					1.314	
Observations					27,990	
ANOVA						
	df	SS	MS		F	Significance F
Regression	4	49,952.066	12,488.017		7231.067	0
Residual	27,985	48,329.954	1.727			
Total	27,989	98,282.020				
	Coefficients	Standard Error	t Stat	*p*-value	Lower 95%	Upper 95%
Intercept	−0.879	0.043	−20.612	<0.001	−0.962	−0.795
Initial CVAI	−0.434	0.003	−150.412	<0.001	−0.440	−0.429
Entry Age	0.007	0.000	50.201	<0.001	0.007	0.007
No Torticollis	−0.170	0.018	9.398	<0.001	−0.205	−0.135
Laterality (Left)	−0.357	0.017	−20.752	<0.001	−0.391	−0.324

## Data Availability

The data analyzed in this study is part of Cranial Technologies’ electronic health record (EHR) and is protected by privacy restrictions. The data cannot be made publicly available.

## Abbreviations

The following abbreviations are used in this manuscript:

CRO	Cranial remolding orthosis
IDP	Isolated deformational plagiocephaly
CVAI	Cranial vault asymmetry index
CVA	Cranial vault asymmetry
